# The Perception and Awareness of the Public about Cancer and Cancer Screening in the United Arab Emirates, a Population-Based Survey

**DOI:** 10.3390/clinpract13030064

**Published:** 2023-06-13

**Authors:** Sarah Humaid Al-Shamsi, Abdulla Humaid Al-Shamsi, Mohammed Humaid Al-Shamsi, Adil Sajwani, Mouza Sultan Alzaabi, Omar Al Hammadi, Faryal Iqbal, Humaid O. Al-Shamsi

**Affiliations:** 1 International School of Creative Science, Sharjah P.O. Box 25779, United Arab Emirates; sarah.alshamsi2005@gmail.com (S.H.A.-S.);; 2 Brighton College Dubai, Dubai P.O. Box 48904, United Arab Emirates; 3 Mediclinic Parkview Hospital, Dubai P.O. Box 51122, United Arab Emirates; 4 College of Medicine and Health Sciences, United Arab Emirates University, Al Ain P.O. Box 15551, United Arab Emirates; 5 Al Rahba Hospital, Sheikh Khalifa Medical City, Al Rahba P.O. Box 34555, United Arab Emirates; 6 Burjeel Medical City, Abu Dhabi P.O. Box 92510, United Arab Emirates; 7 Department of Oncology, Burjeel Cancer Institute, Burjeel Medical City, Abu Dhabi P.O. Box 92510, United Arab Emirates; 8 College of Medicine, University of Sharjah, Sharjah P.O. Box 27272, United Arab Emirates; 9 Emirates Oncology Society, Dubai P.O. Box 6600, United Arab Emirates

**Keywords:** survey, cancer, screening, awareness, UAE

## Abstract

The United Arab Emirates (UAE) has one of the fastest growing economies in the world, which has resulted in an increase in the detection of noncommunicable diseases such as cancer. Despite its screening and early detection efforts falling short of the target coverage of the intended population, the number of reported cases and deaths in UAE has increased over the years. A few studies have been conducted to understand the hurdles to cancer screening in UAE, mostly focusing on breast and colorectal malignancies. There are no studies or surveys that have addressed the barriers and hurdles to overall cancer screening in UAE’s population. Through this is the largest survey to date, we aimed at assessing UAE society’s perception of cancer and early screening and detection. The survey was constructed using the SurveyPlanet platform. The survey was then distributed on social media for direct and snowball sampling, including Instagram, WhatsApp, LinkedIn, Meta (Facebook), and Twitter. Interestingly, 71.3% of the respondents reported that they were fine mentioning or discussing cancer, while 28.2% reported that they were not comfortable with it. Moreover, 91.8% of the respondents knew the meaning of the early detection or screening of cancer, while 8.2% did not. The ability of respondents to correctly identify different types of cancer screening varied. This study suggests that regulatory authorities need to raise more awareness about cancer, especially among younger generations, and create screening recommendations and guidelines that should include younger people. Lastly, hospitals, cancer charity organizations, educational institutes, and the media should address target audiences to raise cancer awareness among them.

## 1. Introduction

Cancer continues to be a major health issue in the United Arab Emirates (UAE), accounting for its third leading cause of death [1]. With a projected increase in UAE’s cancer burden of 300% by 2040 [2], early detection and screening remain the most critical weapons in our fight against cancer, both globally and regionally [2]. Despite the availability of cancer screening for breast, colorectal, and cervical cancers in UAE, according to data from UAE Ministry of Health and Prevention, screening uptake remains very low [1]. Such a low uptake can be explained by poor public awareness, difficulties in obtaining access to cancer screening centers, and other social stigmas about cancer [3]. A few studies have tried to address the barriers to cancer screening in UAE, mostly with a focus on breast and colorectal cancers [3,4,5,6]. To our knowledge, there are no studies or surveys that have addressed these barriers to overall cancer screening in UAE’s population, without focusing on a specific cancer. 

With this background, we conducted a survey aimed at evaluating UAE society’s perception of cancer and early screening and detection.

## 2. Methods

The survey was constructed using the SurveyPlanet platform. The survey was then piloted with 10 participants, and feedback was used to clarify some of the questions and make them easier for the participants to understand. The survey was then published for direct and snowball sampling on social media, including Instagram, WhatsApp, LinkedIn, Meta (Facebook), and Twitter. The invitation to participate in the survey made it clear that it was only for UAE residents. The first question in the survey was also used to filter non-UAE residents out of the questionnaire. The participants were also invited to invite others to participate.

## 3. Results

The exact number of invitees is unknown due to the nature of the invitation on social media. The total number of respondents was 2268, with 1378 (60.9%) being female and 886 (39.1%) being male. The age group between 21 and 40 years was the most commonly represented (*n* = 1000, 44.1%), followed by the age groups from 41 to 50 years (*n* = 790, 34.8%), 51–60 years (*n* = 342, 15.1%), >60 years (*n* = 85, 3.7%), and <20 years (*n* = 52, 2.2%) (Appendix A; Q2). Regarding their smoking statuses, 1799 participants (79.7%) were never smokers, 354 (15.7%) were active smokers, and 105 (4.7%) were ex-smokers (Appendix A; Q5). 

### 3.1. Talking about Cancer

A total of 1621 participants (71.3%) reported that they were fine mentioning and/or discussing cancer, while 638 (28.2%) reported that they were not comfortable mentioning or discussing cancer. In total, 9 participants (0.5%) did not respond to this question (Appendix A; Q6). A total of 465 (21.6%) respondents reported they were uncomfortable mentioning cancer because it brought back bad memories of cancer that they would rather forget, either personal or with regard to family members, while 465 (20.9%) respondents reported that they were uncomfortable mentioning cancer because they got anxious about the word, and the remaining 1277 (57.4%) respondents said they did not mind mentioning the word cancer (Appendix A; Q7).

When asked if the respondents knew anyone with a cancer diagnosis, 1346 participants (59.4%) said that they had been diagnosed with cancer or had a relative who had been diagnosed with cancer. A total of 587 (25.9%) respondents reported that someone they knew who was not related to them had cancer, 171 (7.5%) respondents had cancer themselves, and the remaining 161 (7.1%) respondents said they did not know anyone who had cancer (Appendix A; Q8).

### 3.2. General Knowledge about Cancer and Screening 

In evaluating their knowledge about the most common cancers, most respondents (*n* = 1241, 56.1%) correctly identified breast cancer as the most common cancer, while 702 (31.7%) thought colorectal cancer was the most common cancer, and lung cancer was thought to be the most common by 270 (12.2%) respondents (Appendix A; Q9).

When asked if cancer only affects people who have a positive family history with cancer, 1959 (87%) respondents correctly disagreed with this statement, while 293 (13%) people incorrectly agreed with this statement (Appendix A; Q10). When asked about understanding the meaning of the “early detection/screening of cancer”, 2077 (91.8%) responded “yes”, while 185 (8.2%) said “no” (Appendix A; Q11). When asked whether “cancer screening should be only done if there is a family history”, 2017 (89.4%) respondents correctly disagreed with this statement (Appendix A; Q12).

Regarding the age for starting cancer screening, 883 (39%) respondents correctly identified that, for most cancers, cancer screening should start at the age of 40 years if there is no family history, while “any age” was the response with the second most votes, with 439 (19.4%) respondents answering this. The response with the third most votes was “from age 30 years”, with 370 (16.3%) respondents providing this answer. A total of 330 (14.6%) people said they did not know, 154 (6.8%) said people should start at around the age of 20 years, and the remaining 90 said around the age of 50 years (Appendix A; Q13).

A total of 1610 (71.5%) respondents disagreed with the statement that the early detection of cancer is only performed if there are signs and symptoms of cancer, while 643 (28.5%) agreed with the statement (Appendix A; Q14).

### 3.3. Organ-Specific Screening 

A total of 2202 (97.6%) respondents agreed that there is early breast cancer detection screening, while 53 (3.4%) said there is none (Appendix A; Q15). A total of 1528 (68.5%) respondents incorrectly and 703 (31.5%) correctly responded that there is no early stomach/gastric cancer detection screening method (Appendix A; Q16). A total of 1520 (68.3%) respondents correctly identified that there is early lung cancer detection screening, but 706 (31.7%) said that there is none (Appendix A; Q17). A total of 1801 (80.5%) respondents agreed and 435 (19.5%) disagreed that there is early colon cancer screening (Appendix A; Q18). A total of 1349 (60.8%) respondents said that there is early pancreatic cancer detection screening, while the remaining 868 (39.2%) reported, correctly, that there is none (Appendix A; Q19) (Table 1).

### 3.4. Healthcare Providers’ Role in Cancer Screening 

**Figure 1:**
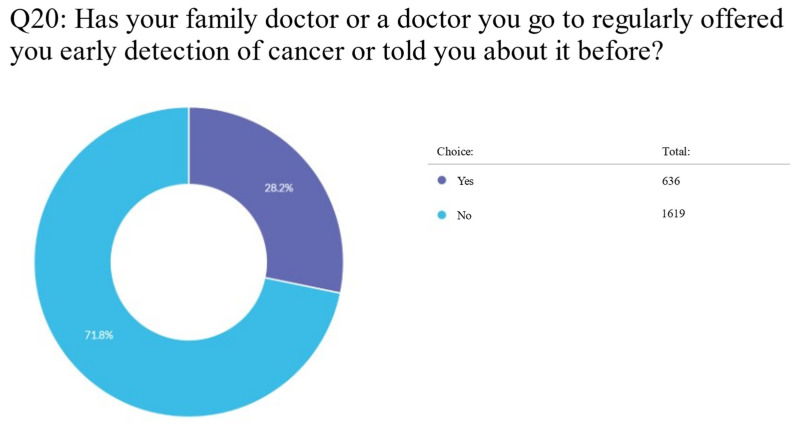
Pie chart of whether participants reported being offered early detection of cancer by their family doctor.

A total of 1619 (71.8%) respondents reported that they had never been informed or invited by their family doctor or the doctor they saw on a regular basis to have an early screening for cancer, or that they had been told about it before, whereas 636 (28.2%) respondents said that they had been told about or offered cancer screening (Appendix A; Q20) (Figure 1). 

**Figure 2:**
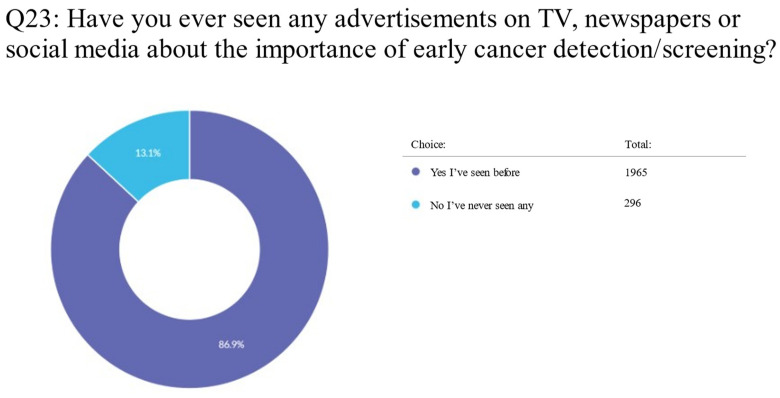
Pie chart on advertisements about the importance of early cancer detection or screening, according to respondents.

For the question of whether their doctor should raise more awareness about the importance of the early detection of cancer, 1942 (86.6%) respondents said “yes”, while 301 (13.4%) said “no” (Appendix A; Q21). For the question of whether they had ever seen any advertisements on TV, in newspapers, or on social media about the importance of early cancer detection or screening, 1965 (86.9%) responded “yes”, while 269 (13.1%) responded “no”, saying they had never seen these advertisements (Appendix A; Q23) (Figure 2).

**Figure 3:**
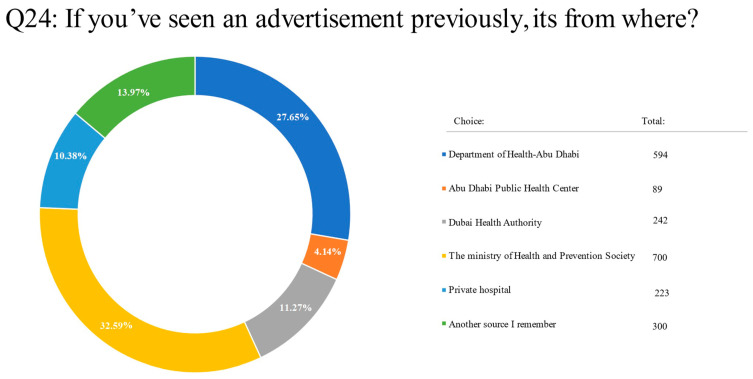
Pie chart on seeing advertisements from a number of healthcare platforms for cancer screening, according to respondents.

A total of 700 (32.5%) respondents had seen a screening advertisement from the Ministry of Health and Prevention, 594 (27.6%) had seen one from the Department of Health Abu Dhabi, 242 (11.2%) had seen one from the Dubai Health Authority, 223 (10.38%) had seen one from a private hospital, and 89 (4.14%) had seen one from the Abu Dhabi Public Health Center. In addition, 300 (13.9%) respondents said they had seen advertisements from other sources (Appendix A; Q24) (Figure 3).

A total of 2159 respondents (96.8%) suggested that there should be continuous media coverage to raise awareness about cancer and the early detection of cancer, and 71 (4.2%) said there should not be (Appendix A; Q26). When asked “do we need to change our mindset about cancer & early detection and screening?”, 2074 participants (91.9%) agreed with this statement and 152 (6.7%) were not sure, while 31 (1.4%) respondents disagreed.

### 3.5. Screening Program Awareness

**Figure 4:**
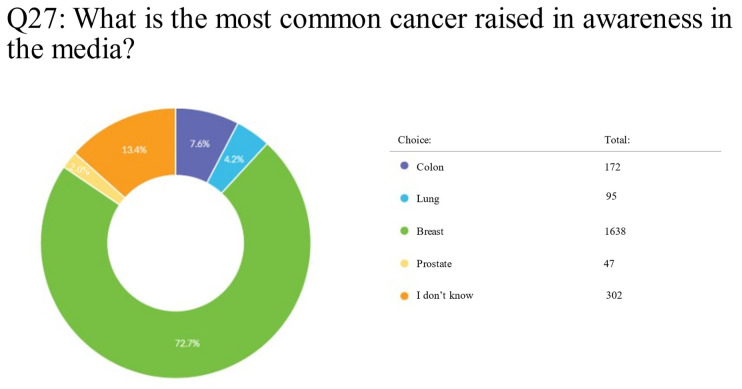
Pie chart of the most frequently mentioned cancers in the media in UAE, according to respondents.

The survey takers had to choose what was the most common cancer receiving awareness in the media, and the results were: breast cancer with a total of 1638 (72.7%) votes, colon cancer with a total of 172 (7.6%) votes, lung cancer with a total of 95 (4.2%) votes, and prostate cancer with a total of 47 (2.1%) votes, while the remaining respondents were not sure (*n* = 302, 13.4%) (Appendix A; Q27) (Figure 4).

We also asked if there are any free programs for UAE citizens for the early detection of cancer, and 1462 participants (65.3%) responded “yes”, while 776 (34.7%) responded that there were none according to their knowledge (Appendix A; Q28). 

Regarding whether there are any free programs for residents (non-citizens) for the early detection of cancer, 896 respondents (40.2%) reported there is a free program for residents and 1332 (59.8%) said that, to the best of their knowledge, there is none (Q 29).

When asked if “early diagnosis by early detection of cancer increases the chances of cancer cure”, 2128 (94.4%) respondents answered with “yes”, 21 (1.3%) answered “no”, and 106 (4.7%) respondents stated they were not sure (Appendix A; Q30).

### 3.6. Barriers to Cancer Screening and How to Improve Them

**Figure 5:**
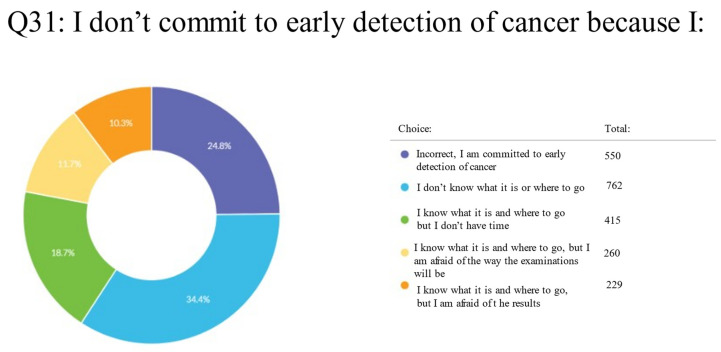
Pie chart of UAE residents on the reasons for not committing to early cancer detection.

When asked about reasons for not adhering to the early detection of cancer, 762 (34.4%) respondents reported that they did not know what or where to go for this early detection of cancer, 550 (24.8%) said they were committed to the early detection of cancer, 415 (18.7%) said they knew what it was and where to go but did not have the time to do so, 260 (11.7%) said they knew what it was and where to go but were afraid of the way the examinations would be, and 229 (10.3%) said they knew what it was and where to go but were afraid of the results (Appendix A; Q31) (Figure 5).

When asked “If there is a simple blood test that does not require any fasting or special preparations and detects many types of cancer without endoscopy or X-rays, then I don’t mind if I do the early detection of cancer on an ongoing basis”, 2100 (93.6%) respondents agreed with this statement, while 114 (5.1%) respondents said they were not sure about it and 29 (1.3%) respondents disagreed (Appendix A; Q32).

When asked if they had previously had any early detection of cancer and its required tests, a total of 893 (42.2%) respondents answered that they were under 40, so they could not answer this, 619 (29.2%) respondents answered that they were above 40 and had undergone the required tests for the early detection of cancer before, and 606 (28.6%) answered that they were above 40 and had not taken the tests for the early detection of cancer before (Appendix A; Q33).

When asked “which of the screening tests have you done before?”, 1191 participants (54.2%) answered that they had not had any previous tests for the early detection of cancer. Mammograms were the most common cancer screening test used by the survey respondents, with 494 people having experienced one (22.5%). A total of 281 (12.8%) respondents reported that they had taken the Pap smear for the early detection of cervical cancer, while 145 (6.6%) reported that they had undergone an endoscopy or occult stool examination for the early detection of colorectal cancer, 67 (3%) had experienced prostate-specific antigen (PSA) testing for prostate cancer, and 21 (0.9%) respondents had computed tomography scans performed for lung cancer screening in smokers (Appendix A; Q34).

Lastly, the respondents were asked if they agreed with the following statement: “The UAE society needs more awareness about cancer, its causes, ways to prevent it, and early cancer detection methods”, and 2194 participants (97.4%) agreed, while 59 (3.6%) disagreed (Appendix A; Q35).

## 4. Discussion

This is the first and largest survey addressing the knowledge and attitudes toward cancer screening in UAE’s population. Previous surveys have only focused on specific and common cancers such as breast cancer and colorectal cancer. In this large survey, both UAE citizens and non-UAE citizens (residents) were invited to participate. Previous surveys have mostly been performed with direct recruitment from clinics and healthcare settings [3,4,5,6]. We utilized social media for our direct recruitment and the snowball effect to obtain a larger sample. The age distribution of our respondents reflected the demographics of UAE’s population. The populations of the age groups of 25–54 and 55–64 years constitute 6.6 million (66.35%) and 0.5 million (5.8%), respectively, in UAE. The country has 0.1 million people over the age of 65 [7].

In contrast to popular belief, our findings show that the majority of people are fine with mentioning and/or discussing cancer, indicating a significant shift from our experience of not being willing to talk about cancer, to being willing to discuss it. There are no older studies or surveys on the previous level of willingness to talk about cancer to compare with the current findings. In evaluating the responses to the general knowledge about cancer and screening, the responses indicated a good general knowledge about the most common cancers, the role of family history, the need for screening irrespective of family history, and the presence of symptoms. Regarding the age to start cancer screening, around 39% of the respondents correctly identified that cancer screening should start, for most cancers, at the age of 40 years if there is no family history, which indicates a greater focus on the awareness about when to start this screening.

When there is no population-based screening program in place, healthcare providers play an integral part in raising awareness and inviting participants to cancer screening, which is a less effective alternative screening [8]. While the majority of respondents in this survey mentioned that they had never been informed or invited by their family doctor or the doctor they saw on a regular basis to have an early detection of cancer, this should be interpreted with caution, because a considerable number of the respondents were not at screening age.

Social media and technology can do wonders when questions about cancer awareness come in. There are many apps and campaigns that have been launched recently for breast cancer, including “Pink Shield”, launched by Emirates Health Services. Since October is breast cancer awareness month worldwide, UAE’s government, as well as private entities, ensure that maximum awareness is raised among women during this month. In accordance with this, the Abu Dhabi Public Health Centre has teamed up with the global biopharmaceutical company MSD and Deliveroo (a food delivery company) to raise breast cancer awareness. These initiatives are being implemented to promote national attempts to fight breast cancer within the scope of the national policy for reducing cancer mortality rates [9,10].

Federal and private initiatives at UAE’s public level have induced excellent awareness among people, especially the younger generation, since they have a better understanding of new technologies. However, private health organizations in UAE still need to make more efforts to raise cancer awareness.

Our survey also shows, beyond a doubt, that the public has an interest in knowing about cancer, especially through continuous media coverage to raise awareness about cancer and the available programs for the early detection of cancer in the country. UAE’s regulatory authorities are spearheading efforts to build effective cancer screening programs in the country. In 2009, a screening program was launched that recommended all female UAE citizens aged 40 years and over to have annual mammography screenings. Following that, in July 2010, a nationwide colorectal cancer screening program was initiated, and by 2014, three screening programs for breast, colorectal, and cervical cancers had been developed [11,12]. Lastly, with the release of lung cancer data in 2017, low-dose computed tomography scans were used to screen for lung cancer [11,13]. Furthermore, several cancer screening and awareness programs have been launched, such as the “Pink Caravan” event. This annual awareness program, which is aimed at raising breast cancer awareness and encouraging screening, now reaches over 45,000 women in UAE [11,14]. In this survey, we also assessed our population’s knowledge about any free programs for citizens for the early detection of cancer. It was apparent that the UAE residents were less aware of free cancer screening and detection programs than the UAE citizens. 

Our survey also showed that, despite the fact that people understand the benefit of early detection in cancer management, a considerable proportion do not know what screening methods are available and where to access them. These results are very significant in terms of addressing the critical issues in cancer screening and early detection pathways. Nothing has a greater impact on the cancer survival rate than screening and early detection. Early cancer detection is thought to increase the likelihood of successful treatment and a better outcome [2]. A dedicated monitoring task force should be established to create a clear pathway for people to let them know where and how to go for cancer screening and should encourage the recruitment of dedicated staff by health organizations that can answer their queries through phone calls or in person. The majority of the people in this survey were hesitant to commit due to the nature of the examination or their fear of the results. A study [15] conducted in Europe also explained these cancer fears. The different facets of cancer fear influence the choice and action processes that lead to screening participation in various ways. Understanding the many behavioral implications of cancer fear may contribute to the development of successful public health messages. The findings of this study suggest that, depending on the component of this fear, cancer fear can be a facilitator or a hindrance. 

Learning more about the components of cancer fear and performing more detailed evaluations of its behavioral consequences may aid in the development of successful public health messages in UAE’s healthcare system, too. Our survey illustrates that people are willing to undergo early detection tests when there are simpler alternatives. 

According to another local survey, four out of every ten women over the age of 40 have never undergone breast cancer screening. The results of this survey were almost consistent with our study. The primary reasons for this hesitancy were a lack of symptoms (31%), being concerned about the results (26%), having no family history of breast cancer (25%), and feeling uncomfortable [16].

## 5. Conclusions

The aim of this largest survey to date was to assess the perception and awareness of cancer and cancer screening in the United Arab Emirates. There are few strengths and limitations of this study, including the fact that an online data survey means a controlled cost methodology and faster data collection, whereas the biggest limitation during the survey was the sample size of the population. 

The goal of cancer screening is to detect pre-cancer or early-stage cancer in asymptomatic patients so that an earlier diagnosis and treatment can be provided, which can result in better outcomes for some people [17]. This survey was designed to be a quick reference that contains the important subjects of the perception and awareness of cancer screening in UAE’s population. It is anticipated that this study will help policymakers to determine whether there are enough cancer screening and awareness programs in UAE to minimize cancer incidence and mortality. There are several barriers to low cancer screening programs in UAE’s population and regulatory authorities are acting or planning to reform these early cancer screening and detection systems (Table 2) [2]. This survey focuses on key messages for policymakers, including the significance of investing in primary prevention and early detection screening programs. Regulatory authorities should use their available resources to improve cancer screening in order to save the lives of their population.

## Figures and Tables

**Table 1:** Survey of different types of cancer detection screening knowledge among participants.

Cancer Type	Correctly Identified If Screening Is Indicated	Incorrectly Identified If Screening Is Indicated
Breast	2202 (97.6%)	53 (2.3%)
Colorectal	1801 (80.5%)	435 (19.4%)
Lung	1520 (68.2%)	706 (31.7%)
Pancreas	1349 (60.8%)	868 (39.1%)
Stomach	1528 (68.4%)	703 (31.5%)

**Table 2:** Barriers and planned activities for promoting early cancer screening in UAE [2].

1. Service Accessibility *Primary Causes* ▪ Access to services is difficult ▪ Cost ▪ The location of testing centres ▪ Screening is not covered by health insurance ▪ Changes in the demographic pyramid and population increase ▪ Recommendations and guidelines do not include younger people
*Reformatory Actions* ▪ Mass education and campaigns on cancer screening and early detection can be the most important regulatory action that can be taken ▪ Address the target audiences at educational institutions, the media, and so forth ▪ Launch of mobile screening services such as mammograms, cervical exams, Fecal Immunochemical Test (FIT) tests, and medical advice ▪ Covering the cost with health insurance or providing it for free ▪ Create special screening package pricing ▪ Establish a cancer detection center 2. Quality Control Mechanisms for Cancer Screening Services *Primary Causes* ▪ The devotion and commitment of health officials and political leaders towards targets and indications for screening and early detection programs ▪ The requirement for a cohesive national cancer screening program ▪ Absence of cancer screening programs ▪ Inadequate application of national screening guidelines ▪ The lack of a specialized team to evaluate the quality of services *Reformatory Actions* ▪ Launching a national cancer early detection program that will use a centralized call system and text messaging to reach out to high-risk populations ▪ Developing a system and targets for measuring coverage rate ▪ Creating a link between the Emirates ID and the cancer screening record ▪ All service providers are committed to meeting the goal percentage ▪ Activating the nationwide screening and early detection registry ▪ Bringing primary care and screening together ▪ Increasing capacity and logistics resources ▪ Appointing quality assurance coordinators ▪ Creating screening services ▪ Launching a quality assurance and monitoring department for cancer screening services ▪ Insurance policies that include screening and early detection ▪ Monitoring the effect of screening on mortality outcomes 3. Community Awareness and the Need for Early Cancer Detection and Screening *Primary Causes* ▪ Inadequate public awareness initiatives ▪ Lack of cancer education and comprehension ▪ Limited incorporation of cancer information into educational curriculum ▪ Insufficiency of smart awareness applications ▪ Lack of understanding of early cancer detection programs ▪ Lack of knowledge about worldwide cancer awareness days for various forms of cancer ▪ Aside from these concerns, various end-user factors influence screening and detection services. Fear, concern about the outcome, shyness, hospital admission, transportation, absence from work, misconceptions and stigma, confidentiality of information, and, last but not least, cultural differences in a society affecting the concepts of prevention and early detection are some of these. *Reformatory Actions* ▪ Performing a cancer awareness measurement assessment across the community using a questionnaire ▪ Increasing awareness efforts about the need for early cancer detection ▪ Details about the availability and location of services ▪ Using prominent and well-known people to raise awareness ▪ Stress the significance of early cancer detection ▪ Create a website for public information ▪ Incorporating cancer education into university/school curriculums and campaigns ▪ Involve representatives from the appropriate authorities ▪ Conducting free cancer detection campaigns during ambulatory care ▪ Smart awareness programs and software to assist community members with making informed decisions and receiving early detection tests 4. Capacity Building in Workforce and Logistics Support *Primary Causes* ▪ Competency deficits ▪ Inadequate human resource distribution ▪ Insufficient logistic resources and sophisticated equipment
*Reformatory Actions* ▪ Strengthening budgetary support for early cancer detection programs ▪ Improving logistical and human resources to expand coverage through manpower and training ▪ Relating general practitioners’ annual performance in primary care facilities ▪ Regularly hosting impartial international experts to evaluate the program and staff ▪ Improving employee efficiency through training courses

## Data Availability

Data can be available from the first author upon reasonable request.

## Appendix A

Appendix A shows the complete cancer survey performed for this study.

**No**	**Question**						
Q2	What is your age?	Below 20	21–40	41–50	51–60	Above 60	
		51	1000	790	342	85	
Q4	Education level?	Middle School	High School	University—Bachelor’s	University—Master’s	University—PhD or higher degree	
		59	478	1224	365	140	
Q5	Do you smoke currently via cigarettes or hookah or medwakh or vape?	Yes		No		I used to, but I quit smoking	
		354		1799		105	
Q6	I don’t like talking about or mentioning the word “cancer”	I don’t mind talking about or mentioning the word “cancer”			I don’t like mentioning the word at all		
		1621			638		
Q7	I don’t like mentioning the word cancer because:	I don’t mind mentioning it		Because I am too anxious to mention it		It brings back bad memories for me or about people I know	
		1277		465		481	
Q8	Do you know anyone who has had cancer in the past or now?	I was diagnosed with cancer	My relative was diagnosed with cancer	Someone I know was diagnosed with cancer		I don’t know anyone who was diagnosed with cancer	
		171	1346	587		161	
Q9	Most common cancer known in UAE?	Lung		Breast		Colon	
		270		1241		702	
Q10	Cancer only affects people that have a family history with cancer	Yes			No		
		293			1959		
Q11	When I hear the sentence “early detection of cancer”, I know what it means	Yes			No		
		2077			185		
Q12	Early detection of cancer is done only when I have a family history of cancer	Yes			No		
		238			2017		
Q13	When should a person, whether male or female, start the early detection of cancer if there is no family history of cancer?	Any age	From the age of 20	From the age of 30	From the age of 40	From the age of 50	I don’t know
		439	154	370	883	90	330
Q14	The early detection of cancer is only done if there are signs. For example, a mass in the breast or pain in the stomach; then we can proceed with colon or breast detection for cancer	Yes			No		
		643			1610		
Q15	Is there early breast cancer detection screening?	Yes, there is			No there isn’t		
		2202			53		
Q16	Is there early stomach/gastric cancer detection screening?	Yes, there is			No there isn’t		
		1528			703		
Q17	Is there early lung cancer detection screening?	Yes, there is			No there isn’t		
		1520			706		
Q18	Is there early colon cancer detection screening?	Yes, there is			No there isn’t		
		1801			435		
Q19	Is there early pancreatic cancer detection screening?	Yes, there is			No there isn’t		
		1349			868		
Q20	Has your family doctor or a doctor you go to regularly offered you an early detection of cancer or told you about it before?	Yes			No		
		636			1619		
Q21	Do you think that your doctor should raise more awareness of the importance of early detection of cancer?	Yes			No		
		1942			301		
Q22	We have completed half of the questions and we will continue with the other half. Are the questions simple and smooth till now?	Yes			No		
		2134			123		
Q23	Have you ever seen any advertisements on TV, in newspapers, or on social media about the importance of early cancer detection/screening?	Yes, I’ve seen before			No, I’ve never seen any		
		1965			296		
Q24	If you’ve seen an advertisement previously, where was it from?	Department of Health-Abu Dhabi	Abu Dhabi Public Health Center	Dubai Health Authority	The ministry of Health and Prevention Society	Private hospital	Another source I remember
		594	89	242	700	223	300
Q25	Should we change our mindset in UAE when it comes to publicly addressing and considering the early detection of cancer?	Yes		No		Not Sure	
		2074		31		152	
Q26	I want continuous media coverage to raise awareness about cancer and the early detection of cancer in the country?	Yes			No		
		2159			71		
Q27	What is the most common cancer raised in awareness in the media?	Colon	Lung	Breast	Prostate	I don’t know	
		172	95	1638	47	302	
Q28	Are there any free programs for citizens for the early detection of cancer?	Yes, there is			There is none according to my knowledge		
		1462			776		
Q29	Are there any free programs for residents for the early detection of cancer?	Yes, there is			There is none according to my knowledge		
		896			1332		
Q30	Early diagnosis via the early detection of cancer increases the chances of obtaining better results and chances of recovery	Yes		No		Not Sure	
		2128		21		106	
Q31	I don’t commit to the early detection of cancer because I:	Incorrect, I am committed to the early detection of cancer	I don’t know what it is or where to go	I know what it is and where to go but I don’t have time	I know what it is and where to go, but I am afraid of the way the examinations will be	I know what it is and where to go, but I am afraid of the results	
		550	762	415	260	229	
Q32	If there is a simple blood test that does not require any fasting or special preparations and detects many types of cancers without endoscopy or x-rays, then I don’t mind if I do the early detection of cancer on an ongoing basis	Yes, I agree totally		I don’t agree		Not sure	
		2100		29		114	
Q33	Only if you are above 40 can you answer this question. Have you done any early detection of cancer and required tests before?	I am under 40 years old		I am above 40 and I have done the required tests for the early detection of cancer before		I am above 40 and I have not done the required tests for the early detection of cancer before	
		893		619		606	
Q34	I did previous tests for the early detection of cancer	Mammogram for the early detection of breast cancer	Endoscopy or occult stool examination for the early detection of colon and rectal cancer	Pap smear for the early detection of cervical cancer	Blood test via *PCR* for prostate cancer in men	CT scan for smokers for lung cancer	I haven’t done any of them before
		494	145	281	67	21	1191
Q35	The last question, UAE’s society needs more awareness about cancer, its causes, ways to prevent it, and early cancer detection methods	Yes, I agree			No, I don’t agree		
		2194			59		
